# Perspectives of Older Adults With and Without Cognitive Impairment on GPS‐based Driving Monitoring Systems: An Exploratory Study

**DOI:** 10.1002/alz70858_106074

**Published:** 2025-12-26

**Authors:** Ranuki Onara Hettiarachchige, Mark J. Rapoport, Gary Naglie, Christopher Pilieci, Shahab Alizadeh, Sayeh Bayat

**Affiliations:** ^1^ University of Calgary, Calgary, AB, Canada; ^2^ University of Toronto, Toronto, ON, Canada; ^3^ Sunnybrook Health Sciences Centre, Toronto, ON, Canada; ^4^ Baycrest Health Sciences, Toronto, ON, Canada; ^5^ Toronto Rehabilitation Institute, Toronto, ON, Canada; ^6^ Rotman Research Institute, Toronto, ON, Canada; ^7^ Kunin‐Lunenfeld Centre for Applied Research & Evaluation, Baycrest Academy for Research and Education, Toronto, ON, Canada; ^8^ Hotchkiss Brain Institute, Calgary, AB, Canada; ^9^ KITE ‐ Toronto Rehabilitation Institute, University Health Network, Toronto, ON, Canada

## Abstract

**Background:**

Dementia impairs cognitive functions, potentially compromising an individual's ability to drive safely. GPS‐based Driving Monitoring Systems (DMS) are increasingly used to study naturalistic driving patterns of individuals with cognitive impairment in real‐world conditions. However, the successful implementation of DMS technologies depends on a comprehensive understanding of their acceptability and usability.

**Objective:**

To evaluate the usability and acceptability of DMS among cognitively intact older adults and older adults with mild cognitive impairment (MCI).

**Method:**

Thirteen participants (9 cognitively intact, 4 with MCI) had a DMS installed in their vehicles for 8 weeks. On the last day, participants completed a user acceptance questionnaire, which included items on *Perceived Usefulness* (e.g., “The device could improve the quality of my driving”), *Perceived Privacy* (e.g., “The device threatens my privacy”), *Perceived Comfort* (e.g., “Using the device was annoying”), and *Facilitating Conditions* (e.g., “The device limits how I like to drive”). Responses were measured using a 6‐point Likert scale ranging from 1=“Completely Agree” to 6=“Completely Disagree.” The median (M) and the interquartile range (IQR) were calculated. Group comparisons were performed using the Mann‐Whitney U test.

**Result:**

Participants in both groups largely disagreed with the statements “The device limits the way in which I like to drive” (M=6, IQR=1.5) and “I think using the device was annoying” (M=6, IQR=2.25), indicating high comfort and minimal perceived interference. To a slightly lesser extent, they also disagreed with the statement “I think that the device threatens my privacy” (M=5, IQR=2.5), reflecting low concerns about privacy. Responses regarding the *Perceived Usefulness* of the device were more varied (M=2.5, IQR=3.75), indicating uncertainty about its potential benefits. Participants with MCI expressed less disagreement with the statement “The device limits the way in which I like to drive” compared to controls (*p* <0.05). No other statistically significant differences were observed. Open‐ended responses included: “I didn’t notice the device,” “It should be smaller,” and “The device didn’t change my driving.”

**Conclusion:**

Preliminary results suggest that older adults with MCI consider the DMS technologies to be acceptable and useful. Future research will involve a larger sample size and explore additional factors influencing acceptance and usability.

